# Synthesis and Functions of Ag_2_S Nanostructures

**DOI:** 10.1186/s11671-015-1125-7

**Published:** 2015-11-02

**Authors:** Chunyan Cui, Xiaoru Li, Jixian Liu, Yongchao Hou, Yuqing Zhao, Guocheng Zhong

**Affiliations:** School of Chemistry Science and Chemical Engineering, Qingdao University, No. 308 Ningxia Road, Qingdao, 266071 China

**Keywords:** Ag_2_S nanostructure, Synthesis, Properties, Application

## Abstract

The paper presents a review about synthesis and applications of Ag_2_S nanostructures. As the modern photoelectric and biological materials, Ag_2_S nanomaterials are potentially useful for both structure and function purposes. Ag_2_S is a direction narrow band gap semiconductor with special properties. Ag_2_S nanostructures have been widely researched in chemistry and biochemistry fields because of their unusual optical, electrical, and mechanical properties. It can also be used in many fields, such as photovoltaic cells and infrared detector. In the past few years, Ag_2_S nanostructures have been synthesized by various methods. The article mainly discusses the four types of preparation methods. Moreover, this article shows a detailed review on the new properties, fabrication, and applications of Ag_2_S nanocrystals.

## Review

### Introduction

Nanoparticles are different from molecular and block materials and have many special physical and chemical properties. In the past decades, the synthetic methods and tuning morphologies of single-component nanocrystalline have made great progress. The metal sulfide nanomaterials have attracted widespread attention due to their suitable band gap, easy manufacturing, low cost, and high performance [1–4].

As an important metal sulfide, Ag_2_S is a direction narrow band gap semiconductor (~1.5 eV), and high-absorption coefficient is approximately 10^4^ m^−1^ [5, 6]. It also has good chemical stability and optical properties [7, 8], so it is widely used in various fields, such as semiconductor, photovoltaic cells, infrared detector, and superionic conductor [9–12]. Recently, it also has been used in the photoelectric switch and oxygen sensor at room temperature [13]. So far, Ag_2_S nanoparticles were successfully prepared by microemulsion method, sol–gel method, particles embedded technology template method, etc. [14–17]. But, it often requires strict preparation conditions, time-consuming, energy consumption, and large size distribution in the traditional production methods. Therefore, simple and economic routes of uniform size distribution of Ag_2_S nanomaterials still have challenges.

In this review, we summarized the preparation and properties of Ag_2_S materials as follows.

### Synthetic Methods

Ag_2_S nanostructures have been synthesized by various methods, such as sol–gel method, particles embedded technology template method, etc. Every method has both advantages and limitations. So the preparation methods have still challenges. The new performance of the Ag_2_S nanostructures needs to be further exploded. So, the article summarized four types of preparation methods to expect to provide a little help for the workers who are engaging in this field.

### Formation of Different Forms of Ag_2_S Nanoparticles

**Fig. 1:**
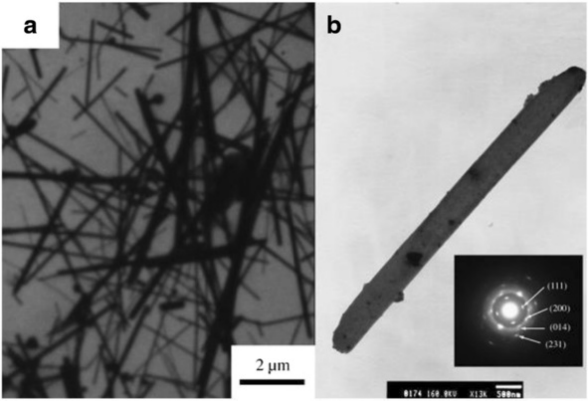
**a** TEM image, **b** SAED pattern of Ag_2_S [23]

**Fig. 2:**
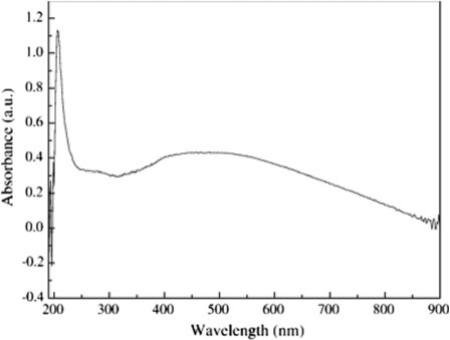
UV–vis absorption spectrum of as-prepared Ag_2_S nanorods [23]

Recently, the great efforts have been focused on the morphology control of the semiconductor nanocrystals due to their morphology-dependent properties [18]. Thus, the preparation of different forms of Ag_2_S nanoparticles (such as urchin-[19], snow-[20], dendrite-like [21, 22] nanocrystals, and so on) has been caused many scientific research in order to expend their current applications. Various methods in the preparation of Ag_2_S nanostructures and their mechanism have been explored. For example, Zhao et al. [23] prepared rod-like Ag_2_S nanocrystallines by using Na_2_S_2_O_3_ as a chalcogen source via gamma ray irradiation at room temperature. In this experiment, polyvinylpyrrolidone (PVP) as a guide reagent of crystal growth plays an important role in the formation of rod-like Ag_2_S nanocrystallines. It is well known that high-energy gamma ray irradiation can make H_2_O producing strong reductive e_aq_^−^ that can initiate many redox reactions to generate ions, which cannot happen in a common atmosphere. And, the homogeneously dispersed S_2_O_3_^2−^ ions reacted with the generated reductive particles to form S^2−^ [24]. And then, Ag^+^ could react with S^2−^ to form Ag_2_S nanoparticles. Ag_2_S nanorods were formed in the solution of the PVP [25]. Figure 1 shows that the diameters of the nanorods rang from 200 to 500 nm, and the length is up to several ten micrometers. The band gap of Ag_2_S nanorods calculated from the UV–vis spectrum is 2.34 eV, which shows an obvious blue shift in UV–vis absorption compared with the bulk Ag_2_S (Fig. 2).

Chen et al. [26] reported that the leaf-like Ag_2_S nanosheets were prepared successfully by a facile hydrothermal method in alcohol–water homogenous medium. In the experiment, CS_2_ used as sulfur source was dissolved in alcohol and AgNO_3_ and water at the beginning, respectively. Then the two solutions were mixed together. The reaction is as follows:

1 $$ 2{\mathrm{NH}}_3+{\mathrm{CS}}_2\to {\mathrm{NH}}_4\mathrm{NHCSSH} $$

2 $$ 2\mathrm{A}\mathrm{g}{\left({\mathrm{NH}}_3\right)}^{+}+{\mathrm{NH}}_4\mathrm{NHCSSH}\to {\mathrm{Ag}}_2\mathrm{S}\downarrow +{\mathrm{NH}}_4\mathrm{S}\mathrm{C}\mathrm{N}+{{2\mathrm{N}\mathrm{H}}_4}^{+} $$

**Fig 3:**
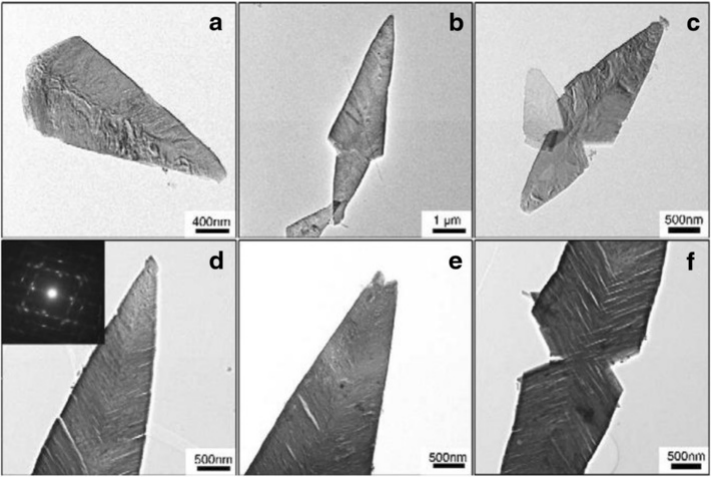
The typical leaf-like morphology TEM image of Ag_2_S nanosheets with different magnification, angle and part prepared in alcohol-water medium by hydrothermal treatment at 160 °C for 10h, the insert in (**d**) shows the SAED spots [26]

The picture of TEM (Fig. 3) shows all of the samples are leaf-like.

**Fig. 4:**
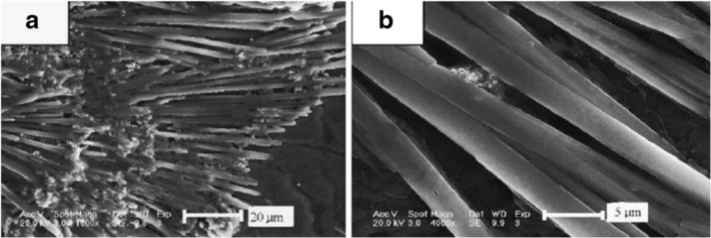
**a** FE-SEM image of spokewise microbars of Ag_2_S, *scale bar*: 20 μm, mole ratio of Ag^+^/Tu: 3/4; **b** Magnified SEM image of the products *scale bar*: 5 μm [30]

**Fig. 5:**
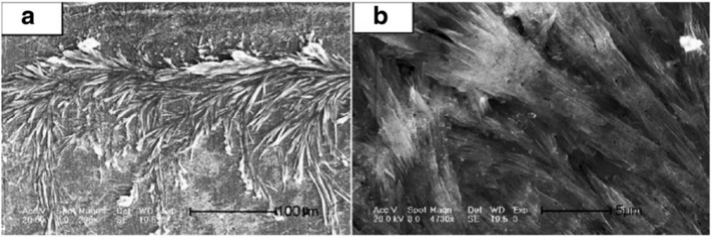
**a** FE-SEM image of microfiber like Ag_2_S, *scale bar*: 100 μm, mole ratio of Ag^+^/Tu: 3/20; **b** Magnified SEM image of the products, *scale bar*: 5 μm [30]

**Fig. 6:**
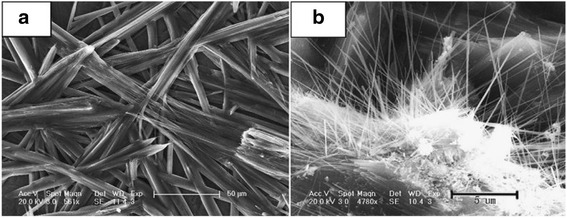
**a** FE-SEM image of the aggregated microwires of Ag_2_S, *scale bar*: 50 μm, mole ratio of Ag^+^/Tu: 3/1; **b** SEM image of Ag_2_S nanowires, *scale bar*: 5 μm [30]

**Fig. 7:**
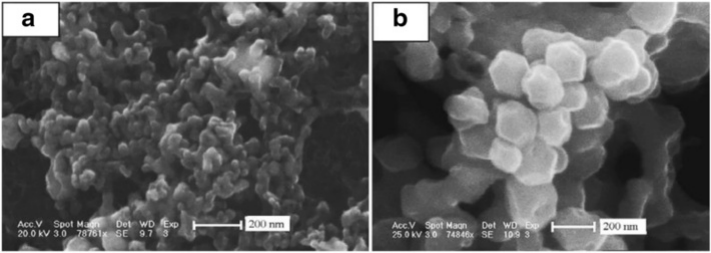
FE-SEM images of Ag_2_S, mole ratio of Ag^+^/Tu: 3/4. **a** worm-like nanoparticles, the initial concentration of ammonia: 7 %; **b** Nanopolyhedrons without ammonia [30]

However, Xu et al. [27] reported that the Ag_2_S was prepared with an alcohol solution method using CS_2_ as sulfur source also. But, all the products were irregular nanoparticles. The reaction medium was changed from water and alcohol–water to alcohol. So, the morphology changed from big irregular nanosheets and microspheres, leaf-like nanosheets nanoparticles to big microspheres, respectively. It is found that alcohol–water homogenous condition and sulfur source of NH_3_-CS_2_ played the key roles in constructing this unique morphology. That is because the formation of nucleation and growth is expected to be strongly dependent on the properties of the solvent during processes such as coarsening and aggregation [28, 29]. The different morphologies of Ag_2_S nano- and micromaterials, including spokewise micrometer bars, nanowires, and nanopolyhedrons have been gained by a facile one-step method at room temperature [30]. In the route, organic template materials were not added into the reaction container. It only changed the ratios of Ag^+^, S^2−^, and ammonia, which may produce the different size and morphologies of the products. In this route, the spokewise microbars of Ag_2_S were successfully prepared. Firstly, 10 mL of 0.2 M Tu ((NH_2_)_2_CS) was added in 10 mL of 0.7 % ammonia. Then, 5 mL of 0.3 M Ag_2_NO_3_ was quickly added in the solution with stirring for 20 min. Last, the product was purified and redispersed in the water several times by centrifugation and sonication (Fig. 4). The methods with other morphologies of Ag_2_S were similar to the above process, except for the concentration of ammonia, AgNO_3_, or Tu. By controlling the concentration of ammonia, AgNO_3_, or Tu, the morphology of Ag_2_S could be easily tuned (Table 1 and Figs. 5, 6, and 7).

**Table 1:** Starting chemicals used in the syntheses of Ag2S and the morphologies of the products [30]

No.	Concentration of reactants	Quantity of reactants (mL)	Morphologies of products
1	Ammonia 0.7 %	10	Spokewise micrometer bars
	AgNO_3_ 0.3 M	5	
	Tu 0.2 M	10	
2	Ammonia 0.7 %	2	Microfibers
	AgNO_3_ 0.06 M	2.5	
	Tu 0.04 M	25	
3	Ammonia 0.7 %	2	Nanowires
	AgNO_3_ 0.06 M	25	
	Tu 0.04 M	12.5	
4	Ammonia 0.7 %	1	Worn-like nanoparticles
	AgNO_3_ 0.3 M	5	
	Tu 0.2 M	10	
5	Without ammonia		Nanopolyhedrons
	AgNO_3_ 0.3 M	5	
	Tu 0.2 M	10

Recently, polyhedral nanocrystals including nanocubes have been attracted intensive attention and a variety of face-centered cubicin organic nanocrystals [31–33] have been successfully fabricated. The Ag_2_S nanocrystals were also prepared by Lim et al. [34] and Wang et al. [35] by decomposing exothermically organometallic precursor silver thiobenzoate (Ag(SCOPh)) and Ag[S_2_P(OR)_2_] (*R* = *C*_*n*_*H*_2*n* + 1_), respectively. However, the method often suffers from elaborate preparation of air sensitive, expensive organometallic complexes that unstable in air. However, the temperature was high during the experimental with the inert gases protecting. And, the solvent was needed to coordinate. The above method has been improved by Dong et al. [36]. A simple hydrothermal route was reported by modulating the ratio of Tu and AgNO_3_ with assistance of cetyltrimethyl ammonium bromide (CTAB), respectively. It was also found that the cooperation effect of CTAB and Tu should be responsible for the formation of the as-obtained Ag_2_S nanocrystals. Face-centered cubic Ag_2_S nanocrystals were synthesized successfully in aqueous medium, which makes the synthesis environmentally benign, user-friendly, economical, and practicable to industry production. The images of SEM showed that the size of the particles ranged from 40 to 80 nm, and the particles with a size of over 100 nm were found occasionally. The typical TEM image showed that most of the Ag_2_S nanocrystals particles appeared hexagonal in shape confirming the faceted nature of the nanocrystal. The UV–vis absorption spectrum of the products showed obvious blue shift owe to the small size.

**Fig. 8:**
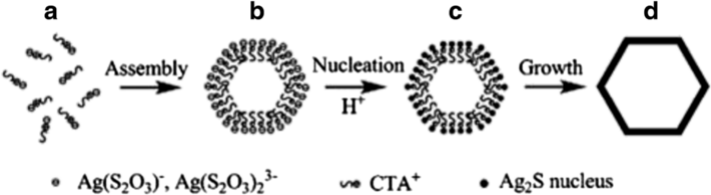
Schematic representation of the formation mechanism of hollow nanohexagons: (**a**) the soluble CTA^+^- Ag(S_2_O_3_) and CTA^+^-[Ag(S_2_O_3_)_2_]^3-^ ion pairs, (**b**) hexagon-like micellar composites with Ag(S_2_O_3_)^-^ and [Ag(S_2_O_3_)_2_]^3-^, (**c**) Ag_2_S nuclei, (**d**) hollow nanohexagon of Ag_2_S [37]

**Fig. 9:**
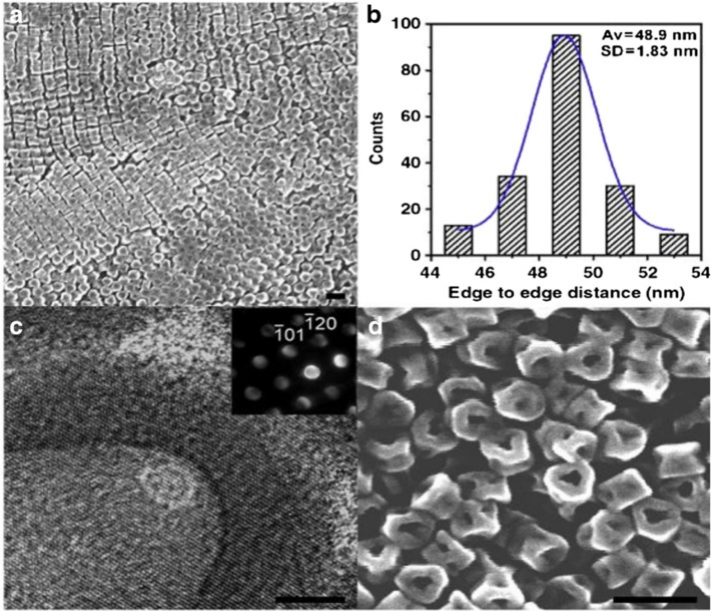
**a** Low-magnification SEM image of hollow nanohexagons, **b** Plane size distribution of hollow nanohexagons, **c** High-magnification TEM image of hollow nanohexagons, and **d** High-magnification SEM image of hollow nanohexagons. The *inset* is the corresponding electronic diffraction pattern from one nanohexagon. *Scale bars*, **a** 100 nm, **b** 5 nm, and **c** 100 nm [37]

**Fig. 10:**
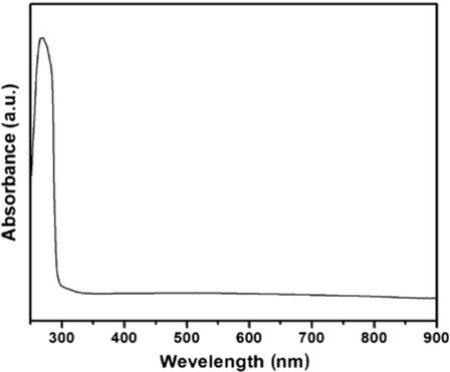
UV–vis absorption spectra of Ag_2_S nanocrystal synthesized in W/O microemulsion ([TAA] = 0.3 mol/L, [Ag^+^] = 0.1 mol/L, [Ag^+^]/[EDTA] = 1) aged for 3d with *ω*_0_ = 10 [41]

**Fig. 11:**
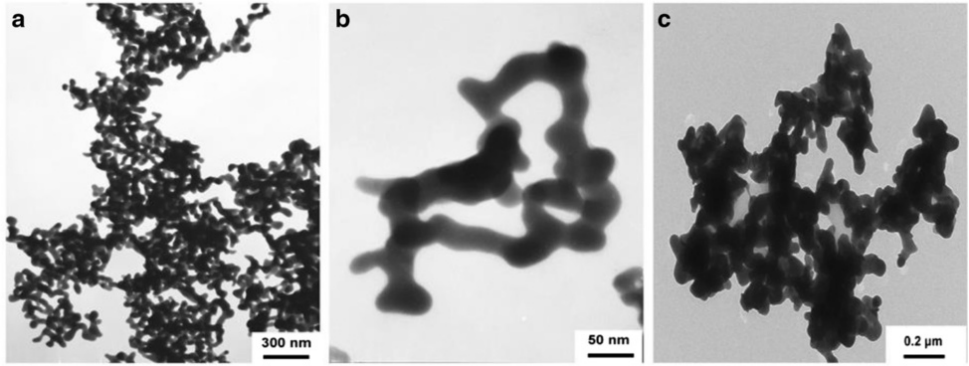
TEM images of Ag_2_S nanocrystal synthesized in W/O microemulsion ([TAA] = 0.3 mol/L, [Ag^+^] = 0.1 mol/L, [Ag^+^]/[EDTA] = 1) **a**,**b** aged for 3d, **c** aged for 24d with *ω*_0_ = 10 [41]

**Fig. 12:**
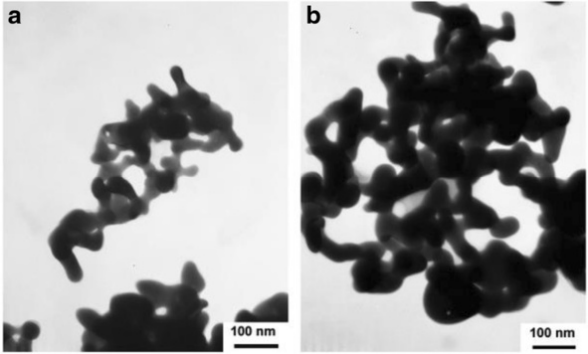
TEM images of Ag_2_S nanocrystal synthesized in W/O microemulsion ([TAA] = 0.3 mol/L, [Ag^+^] = 0.1 mol/L, [Ag^+^]/[EDTA] = 1) aged for 3d with **a** *ω*_0_ = 6, **b** *ω*_0_ = 15 [41]

**Fig. 13:**
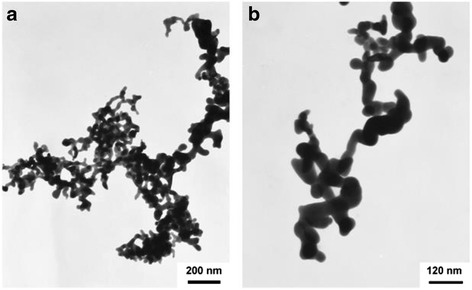
TEM images of Ag_2_S nanocrystal synthesized in W/O microemulsion ([TAA] = 0.3 mol/L, [Ag^+^] = 0.1 mol/L) with various concentrations of EDTA **a** [Ag^+^]/[EDTA] = 2, **b** [Ag^+^]/[EDTA] = 4 aged for 3d with *ω*_0_ = 10 [41]

**Fig. 14:**
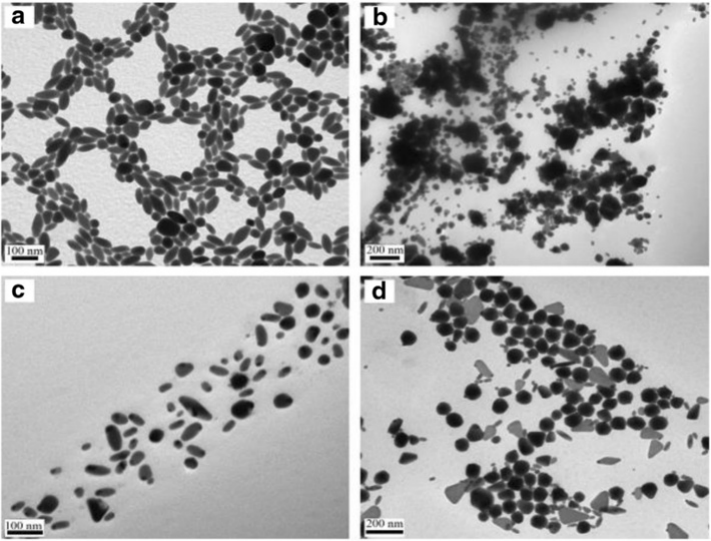
TEM image of Ag_2_S nanoparticles synthesized under different experimental conditions: **a** typical experiment, **b** using AgNO_3_ as the silver source, **c** 160 °C, 4 h, and **d** 160 °C, 13 h[44]

**Fig. 15:**
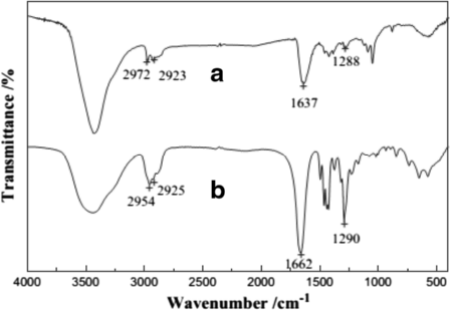
**a** FT-IR spectra of PVP-AgPVP-Ag_2_S, and **b** pure PVP [44]

Ag_2_S nanomaterials were synthesized by a large number of methods. For example, single-crystalline Ag_2_S hollow nanohexagons with better quality and narrower size distribution were successfully reported in aqueous solution at low temperature [37]. The formation mechanism of Ag2S hollow nanohexagons is shown in Fig. 8. Figure 9 displays the SEM images of a typical of Ag_2_S nanohexagons. It indicates that the products are good uniformity. In addition, they are hexagonal in shape with a narrower plane size distribution. And, an edge-to-edge distance of 48.9 ± 1.83 nm was achieved by using this approach. From high-magnification TEM image and high-magnification SEM image show that some of these nanohexagons broke. It is shown that they are hollow inside and single crystalline. Because of the high uniformity and Vander Waals interactions, the hollow nanohexagons spontaneously assemble into high-quality ordered arrays [38, 39]. Due to the character, the hollow nanohexagons may have potential applications in fabricating new useful nanodevices in the future. Meanwhile, Rajib Ghosh Chaudhuri et al. [40] reported an easy and novel route for the synthesis of hollow Ag_2_S particles by a sacrificial core method in surfactant containing aqueous media. High-aspect-ratio of worm-like Ag_2_S nanocrystal with length up to several micrometers and the diameter of 25~50 nm has been successfully prepared by a Triton X-100/cyclohexane/hexanol/water W/O reverse microemulsion in the presence of TAA (thioacetamide) as a sulfur source and EDTA (ethylenediaminetetraacetic acid) as a chelating ligand [41]. The as-synthesized Ag_2_S nanocrystals exhibit strong absorption in UV region, and the absorption edge at about 290 nm (Fig. 10) corresponding to the band gap of 4.3 eV. Compared to the absorption band of bulk Ag_2_S (1240 nm, Eg = 1.0 eV) [42], the observed absorption edge is a significant blue-shift. The result is due to the position-dependent quantum-size effect and shape effect. Figures 11, 12, and 13 show the TEM images of Ag_2_S nanocrystal synthesized with the typical experimental procedure, in which change one condition. The results indicate that the morphology and size of Ag_2_S nanocrystal can be readily controlled by modulating the mole ratio of Ag^+^ to EDTA, the molar ratio of water to surfactant (*ω*_0_), and the aging time. The diameter distribution of Ag_2_S nanocrystal becomes wider with the increasing *ω*_0_. It can be explanted that at low *ω*_0_ water inside the reverse micelles is considered to be “bound”. Therefore, insufficiently available to dissolve the surfactant head group and counterion, the microemulsion becomes more fluid with increasing *ω*_0_, which accelerated the growth of nanocrystalline Ag_2_S. The effect of EDTA concentration on the formation of worm-like Ag_2_S nanocrystals is important. It can coordinate Ag^+^ to form relative stable Ag-EDTA complex, which lowers the effective concentration of Ag^+^; TAA could release S^2−^ very slowly in the solution. The two aspects make the Ag^+^and S^2−^react slowly, which could result in the separation of nucleation and growth step and is favorable for the directional growth of the crystal nuclei [43]. Ag_2_S nanorice was synthesized by reaction between [Ag(NH_3_)_2_]^+^ and Na_2_S·9H_2_O in the presence of PVP through hydrothermal method [44]. And, the feature of the Ag_2_S nanostructure depends mainly on the type of sliver source, influence of the pyrrolidone rings of PVP, reaction time, and temperature. TEM technique was employed to inspect the morphological variation of the Ag_2_S nanoparticles obtained by using different sliver sources (Fig. 14 a and b) at 160 °C for 10 h. It was found that the well-dispersed Ag_2_S nanoparticles presented an approximately uniformed rice-shaped morphology. But when AgNO_3_ was changed into Ag[(NH_3_)_2_]^+^ as the sliver source without a control over the Ag^+^ release rate provided by ammonia complexation during the reaction, the rice-shaped morphological feature of Ag_2_S nanoparticles would not been seen, and anamorphous appearance would be presented instead. So, Ag^+^ concentration had a key impact over the formation of the Ag_2_S nanorice. Similar condition also happened while reaction time influences on the experimental results (Fig. 14 c and d). It can be explained by the famous Ostwald ripening mechanism. The FI-IR spectra of pure PVP, Ag_2_S nanorice-associated PVP are shown in Fig. 15. It is easy to find that the pyrrolidone ring plays an important role in the formation of the Ag_2_S nanorice. Normal and flattened rhombic dodecahedral submicrometer Ag_2_S particles were prepared by adjusting the ratio of Tu to AgNO_3_ and volume of concentrated HCl aqueous solution [45].

### Preparation by Using Bionic Technology

**Fig. 16:**
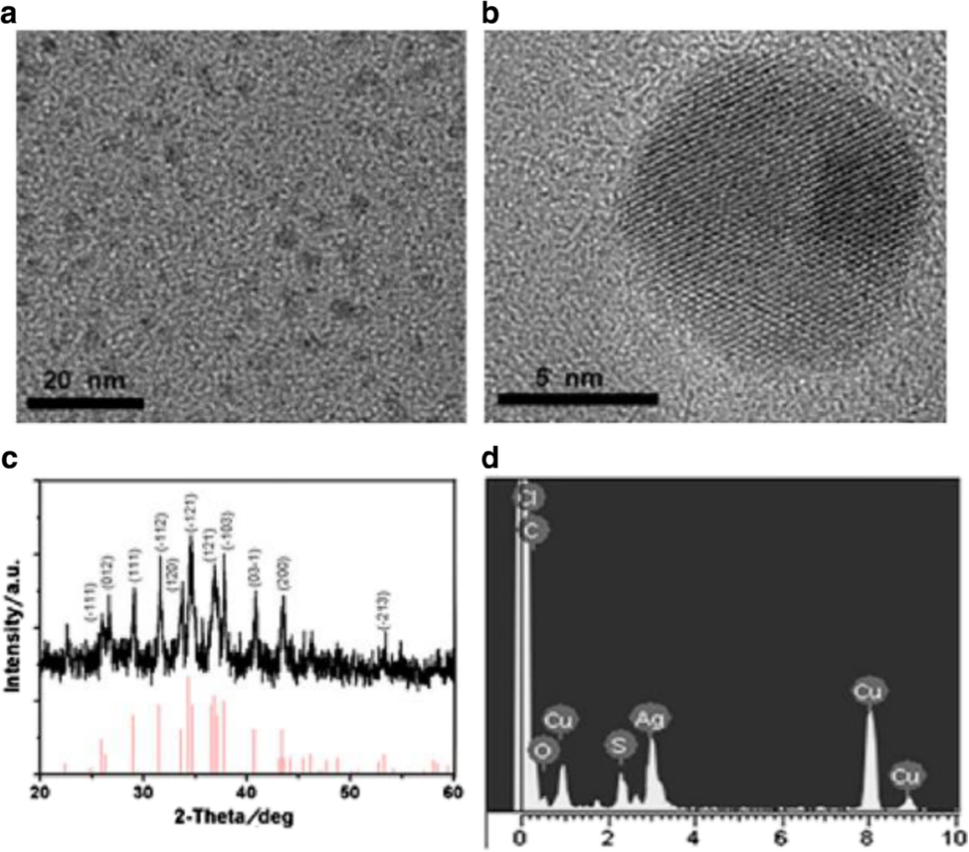
**a** TEM image of fresh prepared RNase A-Ag_2_S QDs. **b** The high-resolution TEM (HR-TEM) image of an individual RNase A-Ag_2_S QD. **c** Powder X-ray diffraction (XRD) pattern of RNase A-CdS QDs. **d** EDS spectrum of RNase A-CdS QDs [51]

In recent years, the synthesis of quantum dots (QDs) with biological macromolecules route has attached great attention [46]. In previous works, CdTe QDs with good biocompatibility by using RNase A as the template was synthesized successfully [48]. But, it is not popular enough because of the toxic nature of Cd and Te. Ag_2_S QDs is treated as an ideal optical probe because it has lower toxicity compared with previous prepared near-infrared (NIR) QDs which was synthesized in organic phase. But, the process may cause extra environment pollution [49, 50]. The synthesis of QDs with fluorescence emission from UV to NIR regions has made great progresses as optical probes for in vitro and in vivo molecular imaging [47]. So, the highly monodisperse and water soluble RNase-Acopped-Ag_2_S QDs clusters were synthesized via biomimetic route in aqueous phase [51]. The QDs have low cytotoxicity and good biocompatibility. Meanwhile, the produce process is environmental friendly. From the images (Fig. 16), it indicates that RNase A-Ag_2_S QDs clusters with irregular morphologies were dispersed, and the Ag_2_S nanocrystals have clear lattice fringes. Furthermore, X-ray diffraction (XRD) image shows that the prepared Ag_2_S QDs assumed the crystalline structure of monoclinic α-Ag_2_S. Tetrazolium-based colorimetric assay (M77 test) shows that RNase A dose not only serves as a stabilizer agent in the formation of Ag_2_S QDs to avoid aggregation but also is a biomolecule to modify the surface of Ag_2_S QDs to decrease toxicity. So, the products have great potential application in molecular imaging in living cells and tissues. Biomolecules assisted the formation of inorganic nanostructures, facilitate the electrostatic stabilization, and thus improving optical properties.

**Fig. 17:**
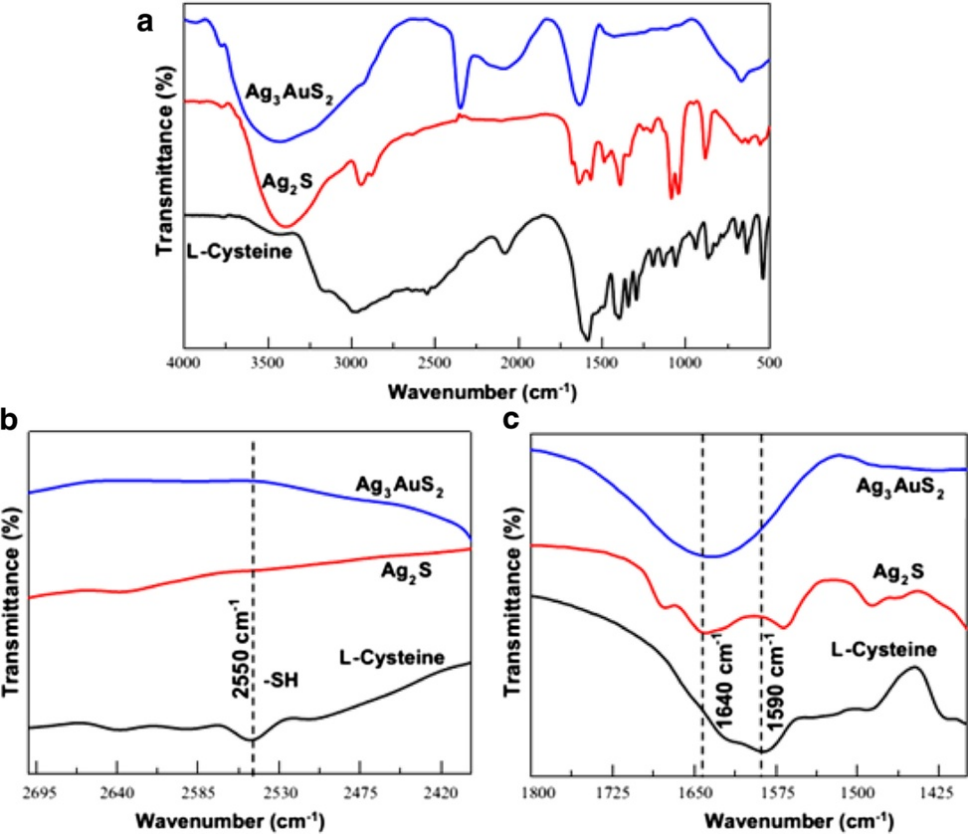
**a** FT-IR spectrum of L-cysteine, Ag_2_S, and Ag_3_AuS_2_ nanocrystals. **b** and **c** are the magnified graphs to show the-SH stretching, and carboxylic group interaction [52]

**Fig. 18:**
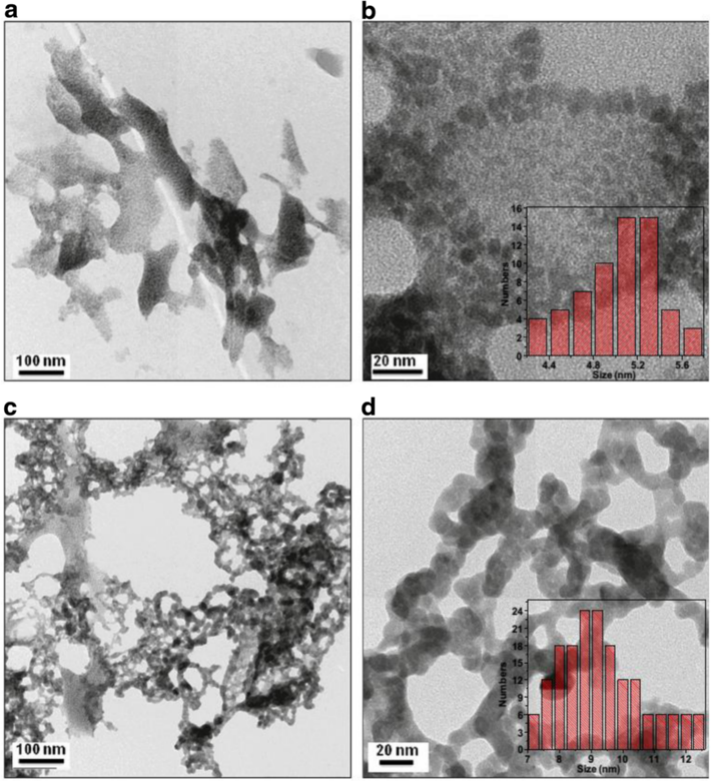
HRTEM images of Ag_2_S (**a**, **b**), Ag_3_AuS_2_ (**c**), and nanocrystals (**d**). *Insets* are the histogram of particle size distribution [52]

Siva C et al. [52] reported that Ag_2_S nanostructure was obtained using Ag^0^ nuclei as a core. Biomolecule (L-cysteine) can act as a sulfur source and stabilizing agent which prevents the agglomeration [53]. Meanwhile, they also have obtained the novel Ag_3_AuS_2_ nanocrystals by adopting the gold ions in the L-cysteine-assisted Ag_2_S formation. The FT-IR images (Fig. 17) show that one L-cysteine molecule is interconnected to another via hydrogen bonding. From Fig. 18, it is clear that the Ag_2_S nanocrystals are almost uniform in their size and are interconnected among themselves, and their average particle size is 5.2 nm. Meanwhile, the Ag_3_AuS_2_ nanocrystals are also connected with themselves. The Ag_3_AuS_2_ nanocrystals are non-uniform in their sizes, and the average size is 9.1 nm. Self-organized nanocrystal architectures with subnanometric spatial resolution also obtained by mimicking the biological crystal growth [54].

### Preparation by a Single Molecular Precursor of Decomposition

**Fig. 19:**
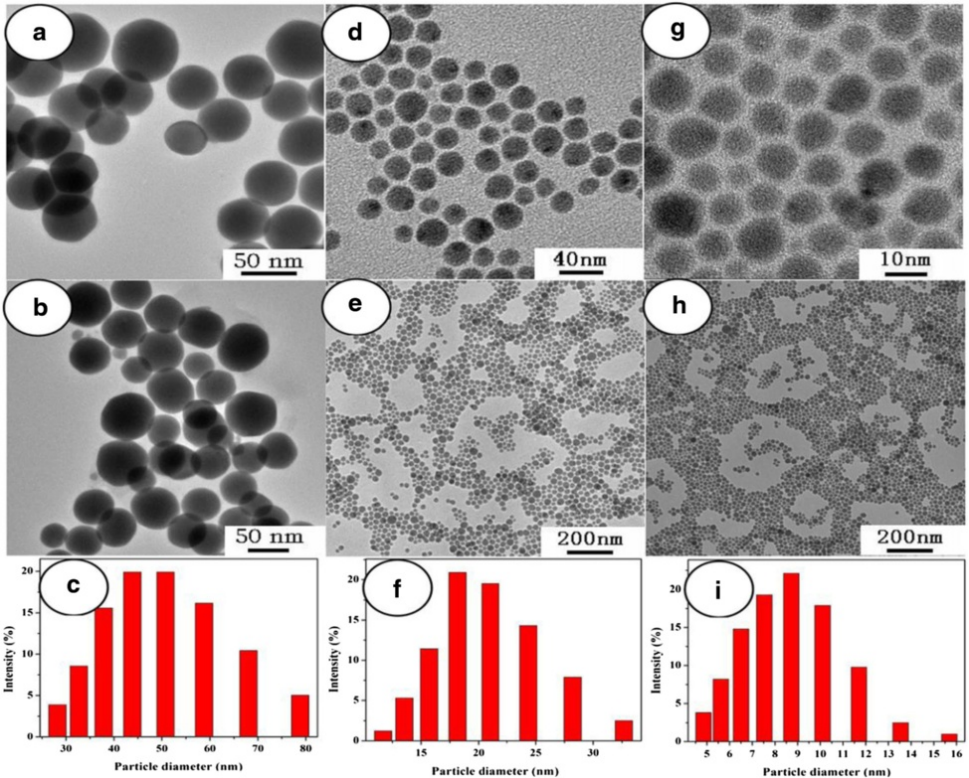
TEM images and corresponding size distribution of Ag_2_S nanoparticles synthesized by solventless thermolysis of silver octyl xanthate (**a**–**c**), silver hexadecyl xanthate (**d**–**f**), and silver carnaubyl xanthate (**g**–**i**) [60]

**Fig. 20:**
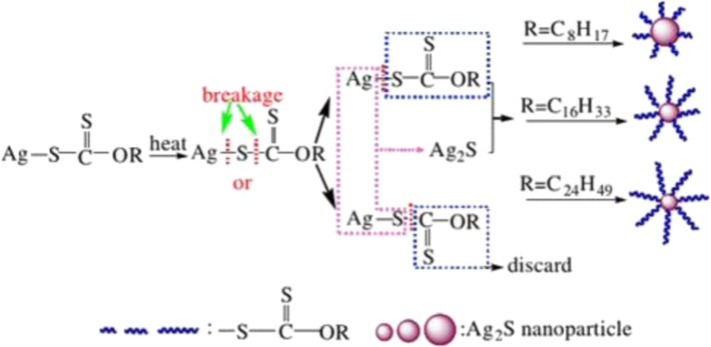
Schematic diagram of the possible growth mode [60]

Among the many methods for synthesizing metal chalcogenide materials, the single molecular precursor route has some appealing features [55, 56]. On the one hand, it offers the distinct advantages of mildness, simplicity, safety, and particular compatibility with the metalorganic chemical vapor deposition [57]. On the other hand, the molecular precursor may be related to the unusual crystal growth selectivity or metastable phase formation of the resultant products, which are sometimes unattainable via conventional synthesis techniques [55, 56]. For example, Ag_2_S nanocrystals were achieved via a modified hot-injection process from a single-source precursor molecule Ag(SCOph). When the precursor molecule is injected into a preheated reaction system at 160 °C, spherical Ag_2_S nanocrystals are directly obtained [58]. Wang et al. [59] obtained the Ag_2_S crystallites by heating the Ag-DDTA in air at 200 °C for 3 h and used an air-stable single-source molecular precursor (Ag-DDTA) as the react source. The method is both economic and non-toxic. Monodispersed and size-controlled Ag_2_S nanoparticles were synthesized successfully via a green and simple surfactant-free solventless thermolysis of silver xanthates as single-source precursors [60]. In the experiment, the diameter of Ag_2_S nanoparticles is from 8.9 ± 1.2 nm to 48.3 ± 3.6 nm (Fig. 19). And, the “Size control” was achieved by simply changing the alkyl chain length in the precursor. The grain size of Ag_2_S nanoparticles decreases with the increase of the alkyl chain length of the precursors. At the same time, with the temperature increasing, the xanthate ligand will be absorbed onto the surface of Ag_2_S nanoparticles to control particle growth. Figure 20 shows the possible growth mechanism.

### Other Methods of Preparation

**Fig. 21:**
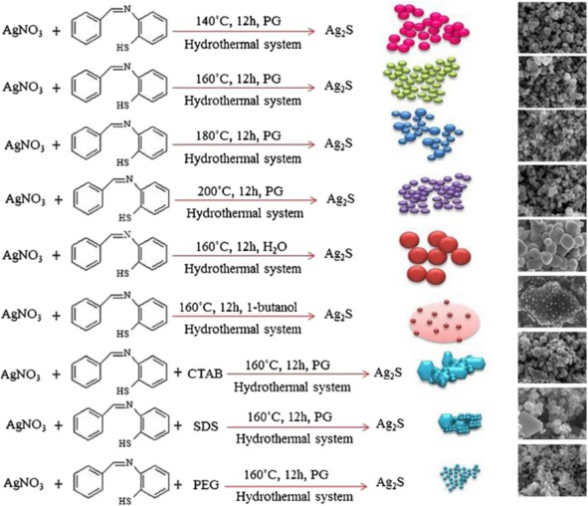
Schematic diagram illustrating the formation of Ag2S samples at various conditions [62]

In addition, there are many other methods. For example, Ag_2_S nanocrystals were prepared via a facile solution growth method, in which Ag_2_S and sulfur powder are used as precursors. Oleylamine is used and act as both reducing agent and stabilizer during the synthetic process [61]. The obtained Ag_2_S nanocrystals can be used as substrates for surface-enhanced Raman scattering (SERS) detection in this method. SERS spectra of rhodamine 6G can be detected, the synthesis strategy is simple, and the obtained samples have great potential for high sensitive optical detection. This character may attract much interest in fundamental physics as well as device application points of view. Maryam Shakouri-Arani et al. [62] have produced the Ag_2_S nanoparticles by a solvothermal process; a new sulfuring agent from class of thio Schiff-base benzenethiol was used in the presence of various solvents. In this paper, we also found that the shape and size of the Ag_2_S can be controlled by means of setting certain reaction parameters such as the reaction temperature, presence of surfactant, and type of solvent (Fig. 21).

## Performance Study

### Application in Biotechnology

**Fig. 22:**
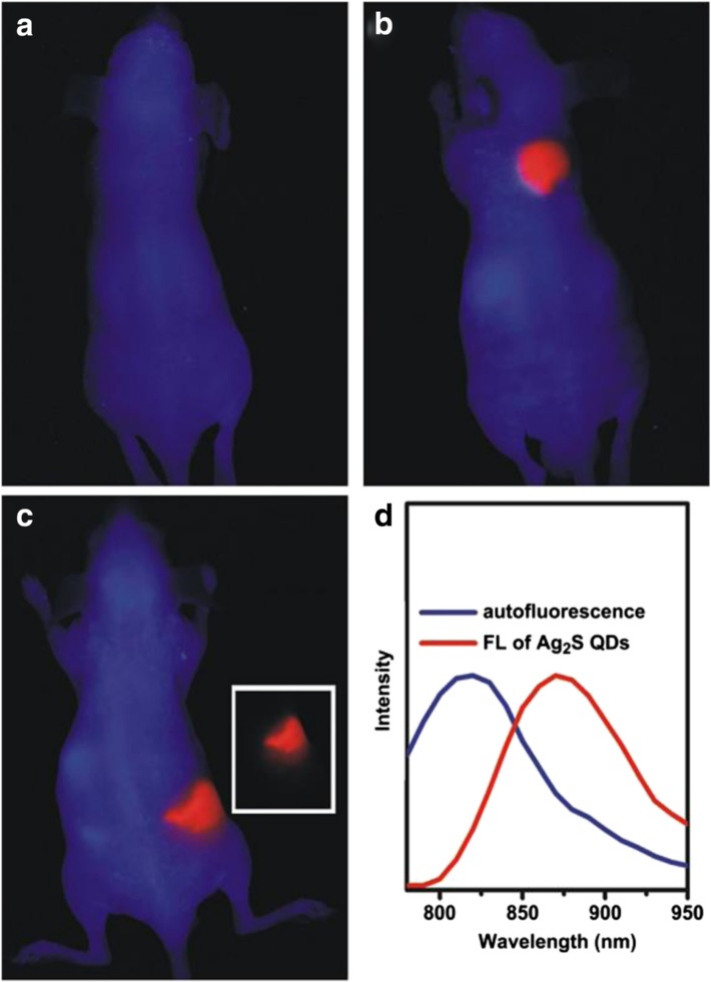
In vivo NIR fluorescence imaging (pseudocolored image) of nude mice. Control experiment (**a**), with subcutaneous injection (**b**) and with celiac injection (**c**) of Ag_2_S quantum dots emitting at 910 nm; Unmixed image of Ag2S quantum dots fluorescence signal (*C inset*); The corresponding emission spectra of the auto fluorescence and QD fluorescence of mice with celiac injection (**d**). (In images **a**–**d**, the *blue* corresponded to the mice auto fluorescence and the *red* corresponded to QD fluorescence.) (For interpretation of the reference to color in this figure legend, the reader is referred to the web version of this article.) [70]

In recent years, Ag_2_S nanometer materials in the application of biotechnology have gradually aroused people’s concern and attention. Many efforts have been devoted to identifying NIR-II emitting agents for in vivo imaging applications. QDs such as PbSe, [63] PbS, [64] and CdHgTe [65], with NIR emission, were successfully obtained. But, the highly toxic nature of Pb, Cd, and Hg is of concern for in vivo applications [66]. And, well-designed carbon nanotubes also have been regarded as biological imaging agent in the NIR-II region. However, the disadvantage is the lower fluorescence quantum yield of carbon nanotubes [67, 68]. So, Zhang et al. [69] made a study for Ag_2_S QDs, which combined with other biomacromolecules to become the imaging agent. Because Ag_2_S QDs should be more biocompatible owning to the absence of any toxic metals such as Cd, Pb, and Hg. And, Ag_2_S also exhibits an ultra-low solubility product constant (Ksp = 6.3 × 10^−50^) which ensures the minimum amount of Ag^+^ released into the biological surroundings. Ag_2_S QDs have high-emission efficiency in the unique NIR-II imaging window. So, there are a lot of characters such as deep tissue penetration, high sensitivity, and elevated spatial and temporal resolution; the water-soluble Ag_2_S QDs terminated with carboxylic acid group were synthesized by one-step method reported [70]. The Ag_2_S QDs exhibited bright photoluminescence and excellent photo stabilities. Therefore, the photoluminescence emission could be turned from visible region to near-infrared (NIR) region (from 510 to 1221 nm). So, it has the opportunity to study nanodiagnostics and imaging. In vivo imaging experiment, the Ag_2_S QDs were injected into the nude mice subcutaneous tissue or abdominal cavity. As shown in Fig. 22a–c, bright spot of Ag_2_S QDs fluorescence was observed in the mice with subcutaneous and celiac injection compared with the ordinary mice. From the PL spectra (Fig. 22d), it can be seen that the fluorescence emitted from the injection region differ with the auto fluorescence from the other region of the mice body. It indicated that the fluorescence of the as-prepared Ag_2_S QDs can penetrate the body of nude mice. And, the fluorescence emitted from the celiac region was clear and bright. It suggested that the Ag_2_S QDs fluorescence was less affected by the body auto fluorescence. At the same time, the Ag_2_S QDs do not contain toxic elements to body. Thus, it has great potential in vivo imaging. And then, Ag_2_S nanocrystals were applied into DNA hybridization analysis [71]. A DNA probe labeled with Ag_2_S nanoparticles, which detection limit can be attained up to picomoles per liter. It indicated that the product have high sensibility and selectivity. Furthermore, this surfactant-capped Ag_2_S product is likely to be of potential application value in electrochemical detection and biosensors.

### Application in the Catalytic and Decomposition

**Fig. 23:**
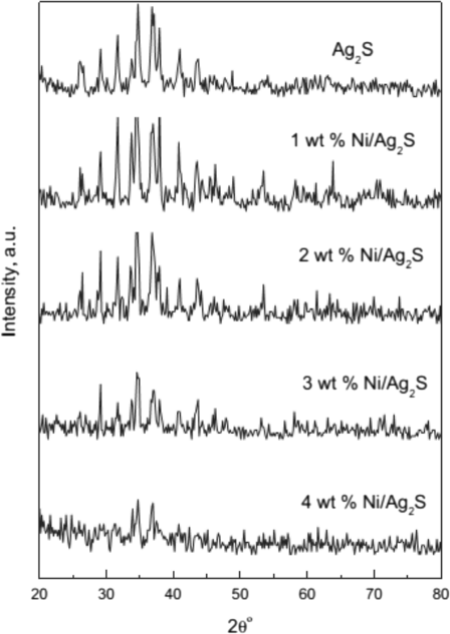
XRD pattern of Ag_2_S and Ni/Ag_2_S nanoparticles [74]

Nowadays, a shortage of clean water can lead to serious problems and diseases. So, water purification problems become more and more important. But, many textile dyes are difficult to degrade with the common methods due to their synthetic origin and the presence of a complex aromatic structure [72, 73] TiO_2_ is often used as catalytic to remove dyes and phenolics for their higher photocatalytic activity, good photo stability, non-toxicity, and low price. However, the large band gap of TiO_2_ (3.2 eV) limits its photocatalytic applications in the UV range and reduces its catalytic efficiency. Because of the unique structure of Ag_2_S, it is expected to be the new type catalyst. And because of the unique structure of Ag_2_S, it can expect to be the new type catalyst. For example, Ag_2_S nanoparticles were prepared by using a hydrothermal method and Ni was doped via a photo-assisted deposition method [74]. The XRD images of the parent Ag_2_S and Ni/Ag_2_S nanoparticles are contrasted in Fig. 23. They found that the structural characteristics of Ag_2_S and Ni/Ag_2_S are mainly composed of Ag_2_S. It indicated that the Ag_2_S structure remained conserved after the application of the photo-assisted deposition methodology. From the UV–vis diffuse reflectance spectra of the Ag_2_S and Ni/Ag_2_S nanoparticles, they calculated that the energy gap decreased with the increasing Ni ions. Ni used as a trapping site captures photo-generated electrons from the conduction band and separates the photo-generated electron–hole pairs. This change would force Ag_2_S to be activated more easily in the visible region, so it can enhance the light absorption ability of the catalysts. And, the catalyst could be reused without any loss in activity for the first 5 cycles. Compared with pure semiconductors, Ag_2_S loaded mesoporous materials in general possess greater photocatalytic activity.

**Fig. 24:**
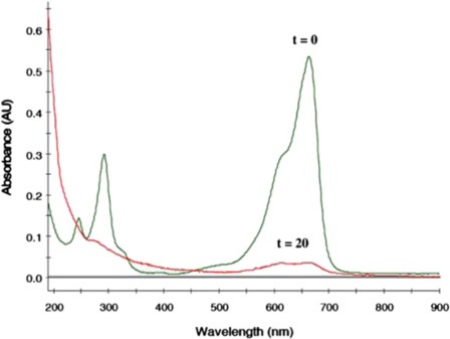
Spectra change that occur during the photocatalytic of aqueous solution of methylene blue: PH = 7. [20 wt% Ag_2_S/MCM = 41] = 0.6 g/L. C_0_ = 0.32 ppm [75]

**Fig. 25:**
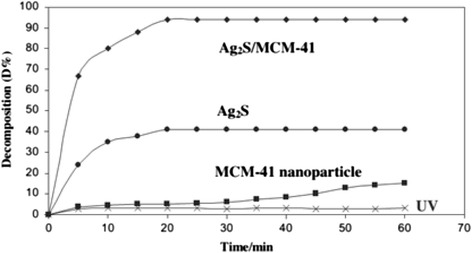
Effect of UV light and different photocatalysts on photocatalystic degradation of methylene blue. C_0_ = 0.32 ppm. [20 wt% Ag_2_S/MCM-41] = 0.6 g/L, PH = 7 [75]

The advantages using zeolite or mesoporous support for semiconductor photocatalysis include formation of ultrafine semiconductor particles during sol–gel deposition, increased adsorption in the pores, surface acidity which enhances electron-abstraction, and decreased UV-light scattering as the main component of zeolite is silica [72, 73]. A. Pourahmad prepared the Ag_2_S/MCM-41 photocatalysts by ion exchange method and is used for the photocatalytic degradation of methylene blue [75]. Figure 24 is the time-dependent electronic absorption spectra of dye during photo irradiation. After 20 min of irradiation under UV light in a Ag_2_S/MCM-41 suspension, 94 % of dye was decomposed and decolorized. And, no new bands appear in the UV–vis region due to the reaction intermediates formed during the degradation process. Under UV irradiation, Ag_2_S, MCM-41, and Ag_2_S/MCM-41 materials on photodegradation of methylene blue are shown in Fig. 25. It is observed that Ag_2_S supported system has a higher rate of degradation than Ag_2_S or MCM-41 alone. And, there are many factors that can influence the efficiency of nanocomposite catalyst, such as the amount of Ag_2_S loading, PH, and initial concentration of dye. Several methods have been reported concerning the photosensitization of TiO_2_ by M_x_S_y_ or M_x_O_y_ nanoparticles for heterogeneous photocatalysis [76] including CdS [77] or WO_3_ [78]. In fact, nanocrystalline Ag_2_S is a good candidate for the photosensitization of TiO_2_ catalysts, for Ag_2_S has a direct band gap of 0.9–1.05 eV, and its conduction band (−0.3 eV) is less anodic than the corresponding TiO_2_ band (−0.1 eV), and the valence band (+0.7 eV) is more cathodic than the TiO_2_ valence band (+3.1 eV). So, distinct TiO_2_/Ag_2_S nanocomposites were prepared by a single-source decomposition method [79]. After, the sensitized TiO_2_ materials were evaluated as photocatalysts on the degradation of aqueous phenol solutions, and the photocatalytic activity of nanocomposites was enhanced with the existence of Ag_2_S over the TiO_2_ surface. And, the efficiency of this photocatalysts is considerably improved comparing with pure TiO_2_. The best phenol photocatalyst was obtained when atomic ratio of Ti/Ag is 2.40. Nanostructured Ag_2_S/CdS was synthesized by two-step precipitation method [80]. And, the composite materials have certain photocatalytic performance. When the concentration of Ag_2_S was 5 % by weight, Ag_2_S/Cds showed the highest photocatalytic activity for hydrogen evolution, with the solar-hydrogen energy conversion efficiency approximately 0.7 %. So, after doped Ag_2_S, the photocatalytic activity of CdS have enhanced obviously.

### Application in Optoelectronic Devices

**Fig. 26:**
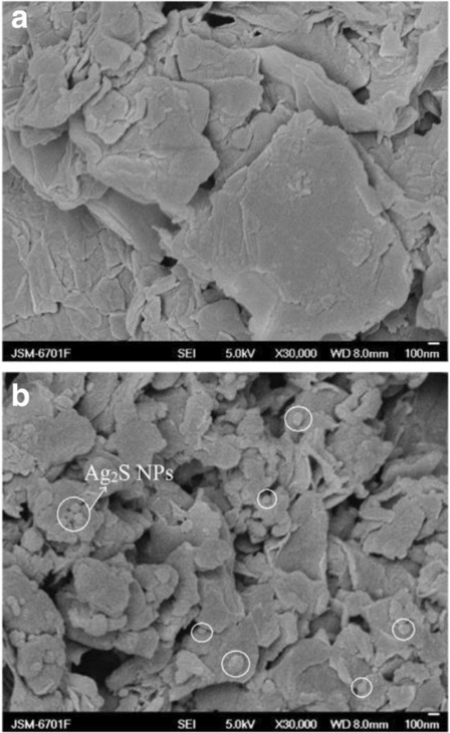
SEM images of **a** the pristine graphene oxide and **b** Gs-Ag_2_S composites [81]

**Fig. 27:**
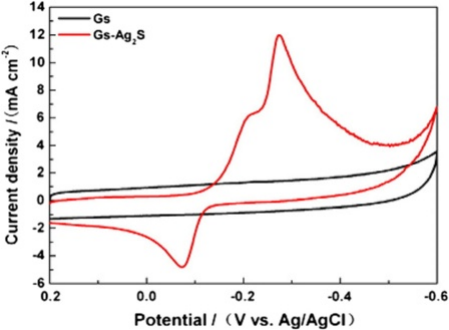
CV curves of graphene and Gs-Ag_2_S composites at 100 mV^−1^ in 1 M H_2_SO_4_ in potential range from −0.6 to 0.2 V [81]

Compared with bulk counterparts, the sheet-like photocatalysts are much better for continuous flow system because of ease separation and recovery from the reaction system [86]. The sheet-like photocatalysts can also help to harvest light more efficiently [87]. So, a novel graphene sheet/Ag_2_S composite was synthesized through a facile solvothermal method, and its electrochemical performance was carried on a modified glassy carbon electrode in a three-electrode electrochemical cell [81]. In Fig. 26, it can be clearly seen that the pure graphene oxide sheets naturally aggregate and stack to multilayers with numerous edges, and the surface of graphene oxide was very smooth compared with graphene sheets doped with Ag_2_S NPs. So, the morphology of G-Ag_2_S composites has a substantial difference from that of the Go sheet. Figure 27 shows that the composite modified GCE shows redox peaks, but graphene modified exhibits a perfect rectangle curve, and the redox peaks of the composites, often a characteristic of pseudocapacitance mainly result from the redox transition of Ag_2_S between a semiconducting state and a conducting state. And, it is calculated that the redox peaks with the specific capacitance is 1063 Fg^−1^, but the specific capacitance of graphene modified is 316 Fg^−1^. So, it is believed that the nanocomposites would be a promising candidate as supercapacitor materials for practical applications in future electronic devices. At the same time, graphene-like Co_3_S_4_ nanosheet/Ag_2_S nanocomposite was prepared using a simple method. The nanocomposite photocatalyst displays excellent stability and photocatalytic activity compared with pure Co_3_S_4_ nanosheet or Ag_2_S nanoparticles [85]. Ag_2_S is an important material for optoelectronic, because it has an energy gap of Eg~1.1 eV, which is similar to the ideal band gap of 1.13 eV for a photovoltaic device [82] indicating that Ag_2_S could be an optimal solar absorber performance which was measured to the battery. The Ag_2_S QDs were synthesized by the successive ionic layer adsorption and reaction deposition method [83]. And, the assembled Ag_2_S-QD solar cell in *λ* = 530 nm has the biggest external quantum efficiency (EQE) which was 59 %, and when the spectral range in 400–1000 nm, the average of EQE was 42 %. The effective scope of photovoltaic is full of visible light and near-infrared spectral regions. Therefore, the results indicate that Ag_2_S QDs can be used as a highly efficient and broad band sensitizer for solar cells. R. Karimzadeh et al. [84] found that about 3 nm Ag_2_S semiconductor nanocrystals in concentrations of dimethyl sulfoxide solution has different non-linear refractive properties; it can be used as a low power optical-limiting device. In addition, Ag_2_S also has important application in other areas, for example nanometer-scale non-volatile memory devices. [88].

### Other Applications

**Fig. 28:**
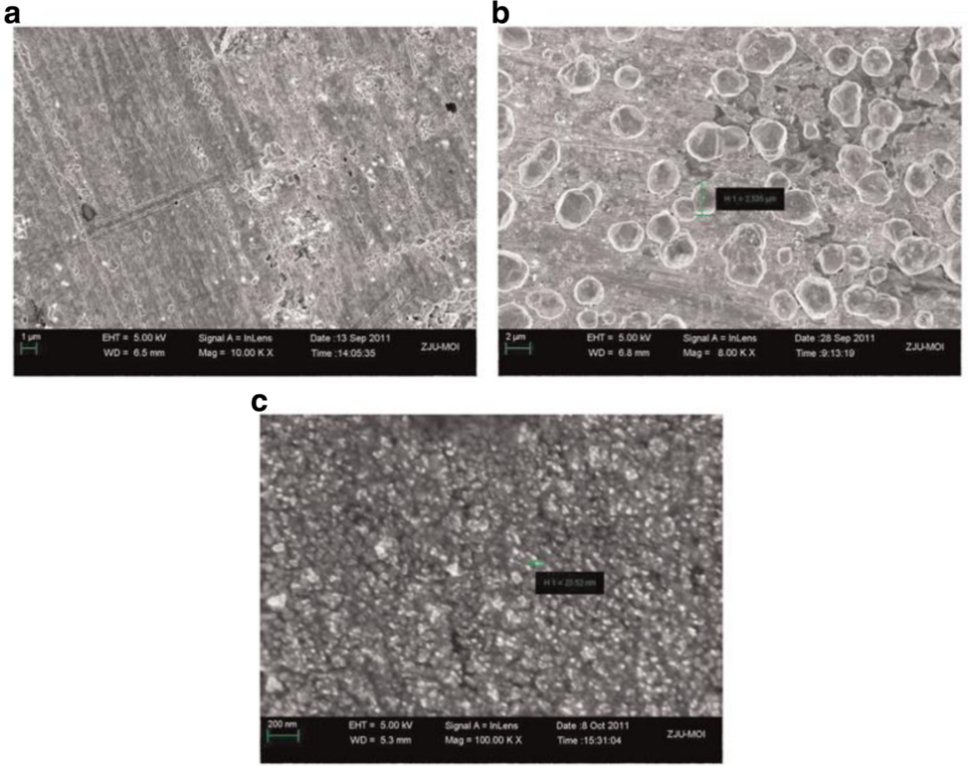
SEM observation of the surface of **a** typical electrode, **b** direct current electroplating electrode, and **c** direct current carrier electroplating electrode [96]

**Fig. 29:**
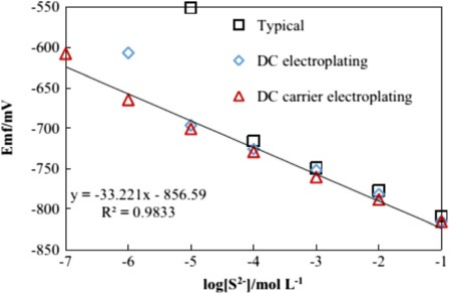
Response curves of the three types of electrode. The linear correlation curve is generated using the direct current carrier electroplating method [96]

Ag_2_S nanomaterials also have many other properties in various fields, such as electronic, magnetic, and so on. Ag_2_S belongs to I-VI semiconductor materials with monoclinic crystal structure. Thin films of Ag2S have applications in photoconducting cells [89], IR detectors [90], and solar selective coating. Thus, Ag_2_S is a promising material for the conversion of solar energy into electricity as its band gap is between 1 and 2 eV. Usually, the material design for these technological applications is based on thin film preparation techniques, in which the film thickness ranges from micrometer to submicrometer. It is well known that the surface contribution to the electric transport process could be evaluated when thin films with different thicknesses, i.e., with various surface-to-volume ratios, are investigated [91]. So, D. Karashanova et al. [92] evaluated the surface contribution to the electronic or ionic transport in the epitaxial silver sulfide films using electron-conducting and electron-blocking contacts, respectively. At the same time, Ag/Ag_2_S also can become the electrode materials, but disadvantages of the solid-state Ag/Ag_2_S electrode such as non-ideal response, signal drifting, and a long response time at low sulfide levels have limited its application [93]. Thus, increasing the precision of the Ag/Ag_2_S electrode by surface micromotion can help the research work under extreme circumstances, such as hydrothermal vents [94, 95]. So, Ding et al. [96] have enhanced the sensitivity of the Ag/Ag_2_S electrode by using direct current carrier power to electroplate silver nanoparticles on a silver wire. Three types of the Ag/Ag_2_S electrode each had different physical structures under SEM (Fig. 28), which indicated that the different surfaces of these electrodes demonstrated that the preparation procedures affected the physical structures of the electrodes. Among all these electrodes, the direct current carrier electroplating electrode has the highest detection limit while the typical electrode has the lowest limit. From Table 2, we can see that the response time of the electrode prepared by direct current carrier electroplating for detecting a concentration of 10^−7^ mol L^−1^ S^2−^ was less than 60 s. And, the detection limits of the Ag/Ag_2_S electrodes prepared by direct current electroplating and direct current carrier electroplating were improved to 1 × 10^−5^ and 1 × 10^−7^ mol L^−1^, respectively. The RMSE (root mean square deviation) of the linear regression for the electrode using the direct current carrier electroplating method verified the accuracy and precision of this type of electrode (Fig. 29). In addition to the above mention, Ag_2_S also has many other properties in applications, but it still needs people to explore it gradually.

**Table 2:** Correlation of EMF (mv) with-log[S^2−^] for the types of electrode [96]

	slope	R2	*n*	RMSE	*p*
Typical	30.81	0.998	4	1.763	<0.001
DC electroplating	29.49	0.998	5	2.231	<0.0001
DC carrier electroplating	33.22	0.983	7	10.24	<0.0001

## Conclusions

Ag_2_S, playing important functions in a number of optical, electrochemical, and biochemical process, has been regarded as a promising sensor and biological imaging agent in living creature. The preparation process and product of Ag_2_S have many disadvantages in traditional preparation methods. For example, it usually needs high temperature, complicated processes, easy gather, and particle size bed control. Recently, considerable efforts have been made to optimize the productive process of Ag_2_S and enhance properties and values of products. This work reviewed the progress in the development of Ag_2_S nanomaterials in the field of synthesis and application. Different forms of Ag_2_S nanostructures have been synthesized such as rod-shaped, leaf-shaped, and cubic. Ag_2_S nanostructure obtained by bionic technology and precursor of decomposition were prepared successfully. Meanwhile, it has been applied to many fields successfully, such as optical, electrical, and biology, and it is expected to use in other fields. In fact, there are still limitations for their practical use in photoelectric and medical fields because it often requires complex preparation process, and the yield is very low. In most cases, Ag_2_S nanoparticles are very prone to gather, which will greatly reduce its optical properties. Therefore, it is often necessary to composite with other materials to achieve a good effect. Although, there are so many challenges, the advances in nanoscience and nanotechnology of Ag_2_S still promise a better future for kinds of industries.

## References

[1] Jun HK, Gareem MA, Arof AK, Sust R (2013). Quantum dot-sensitized solar cells-perspective and recent development: a review of Cd chalcogenide quantum dots as sensitizers. Energy Rev.

[2] Selinsky RS, Ding Q, Faber MS, Wright JC, Jin S (2013). Quantum dot nanoscale heterostructures for solar energy conversion. Chem Soc Rev.

[3] PanL LT, Liu X, Lu T, Zhu G, Sun Z, Sun CQ (2012). Metal-free photocatalytic degradation of 4-chlorophenol in water by mesoporous carbon nitride semiconductors. Catal Sci Technol.

[4] Zhao Z, Liu Z, Miyauchi M (2010). Tailored remote photochromic coloration of in situ synthesized CdS quantum dot loaded WO_3_ films. Adv Funct Mater.

[5] Ezenwa IA, Okereke NA, Egwunyenga NJ (2012). Optical properties of chemical bath deposited Ag_2_S thin films. Int J Sci Technol.

[6] Hwang I, Yong K (2013). Environmentally benign and efficient Ag_2_S-ZnO nanowires as photoanodes for solar cells: comparison with CdS-ZnO nanowires. Chem Phys Chem.

[7] Jiang F, Tian Q, Tang M, Chen Z, Yang J, Hu J (2011). One-pot synthesis of large–scaled Janus Ag-Ag_2_S nanoparticles and their photocatalytic properties. Cryst Eng Comm.

[8] Dlala H, Amlouk M, Belgacem S, Girard P, Barjon D (1998). Structural and optical properties of Ag2S thin films prepared by spray pyrolysis. Eurphys j-appl phys.

[9] Brelle MC, Zhang JZ (1998). Femtosecond study of photo-induced electron dynamics in AgI and core/shell structural AgI/Ag_2_S colloidal nanoparticles. Chem J Phys.

[10] Bruhwiler D, Leigener C, Glaus S, Calzaferri G (2002). Luminescent silver sulfide clusters. J Phys Chem B.

[11] Hull S, Keen DA, Sioia DS, Madden PA, Wilson M (2002). The high-temperature superionic behaviour of Ag_2_S. J Phys Conclens Matter.

[12] Kitova S, Eneva J, Panov A, Haefke H (1994). Infrared photography based on vapor-deposited silver sulfide thin films. Imaging J Sci Fechnol.

[13] Wang DS, Hao CH, Zhong W, Peng Q, Wang TH, Liao ZM, Yu DP, Li YD (2008). Ultralong single-crystalline Ag_2_S nanowires: promising candidates for photoswitches and room-temperature oxygen sensors. Adv Mater.

[14] Liu JC, Raveendran P, Shervani Z, Ikushima Y (2004). Synthesis of Ag_2_S quantum dots in water-in-CO_2_ microemulsions. Chem Commun.

[15] Xiao JP, Xie Y, Tang R, Luo W (2002). Template-based synthesis of nanoscale Ag_2_E (E = S,Se) dendrites. J Mater Chem.

[16] Chen M, Xie Y, Chen HY, Qiao ZP, Qian YT (2001). Preparation and characterization of metal sulfides in ethylenediamine under ambient condition through a γ-irradiation route. J Colloid Interf Sci.

[17] Lim WP, Zhang ZH, Low HY, Chin WS (2004). Preparation of Ag_2_S nanocrystals of predictable shape and size. Angew Chem Int Ed.

[18] Buda C, Chen XB, Narayanan R, Sayed EI (2005). Chemistry and properties of nanocrystals of different shapes. Chem Rev.

[19] Zhang WQ, Xu LQ, Tang KB, Li FQ, Qian WT (2005). Solvothermal synthesis of NiS 3D nanostructures. Eur J Inorg Chem.

[20] Liu QY, Guo F, Komarneni S (2004). Biomolecule-assisted synthesis of highly ordered snowflakelike structures of bismuth sulfide nanorods. J Am Chem Soc.

[21] Kuang D, Xu A, Fang Y, Liu H, Frommen C, Fenske D (2003). Surfactant-assisted growth of novel PbS dendritic nanostructures via facile hydrothermal process. Adv Mater.

[22] Chen XG, Wang X, Wang ZG, Yang XG, Qian YT (2005). Hierarchical growth and shape evolution of HgS dendrites. Cryst Growth Des.

[23] Zhao Y, Zhang DW, Shi WF (2007). A gamma-ray irradiation reduction route to prepare rod-like Ag_2_S nanocrystallines at room temperature. Mater Lett.

[24] Yin YD, XuXG GXW, Lu Y, Zhang ZC (1999). Synthesis and characterization of ZnS colloidal particles via γ-radiation. Radiat Phys Chem.

[25] Chen AH, Wang HQ, Li XY (2005). One-step process to fabricate Ag-polypyrrole coaxial nanocables. Chem Commun.

[26] Chen MH, Gao L (2006). Synthesis of leaf-like Ag_2_S nanosheets by hydrothermal method in water alcohol homogenous medium. Mater Lett.

[27] Xu CG, Zhang ZC, Ye Q (2004). A novel facile method to metal sulfide (metal = Cd, Ag, Hg) nanocrystallite. Mater Lett.

[28] Cheng B, Russell JM, Sheng W, Zhang L, Samulski ET (2004). Large-Scale, solution-phase growth of single-crystalline SnO2 nanorods. J Am Chem Soc.

[29] Wong EM, Bonevich JE, Searson PC (1998). Growth kinetics of nanocrystalline ZnO particles from colloidal suspensions. J Phys Chem B.

[30] Zhai HJ, Wang HS (2008). Ag_2_S morphology controllable via simple template-free solution route. Mater Res Bull.

[31] Gou LF, Murphy CJ (2003). Solution-phase synthesis of Cu_2_O nanocubes. Nano Lett.

[32] Lee SM, Jun Y, Cho SN, Cheon J (2002). Single-crystalline star-shaped nanocrystals and their evolution: programming the geometry of nano-building blocks. J Am Chem Soc.

[33] Seo WS, Shim JH, Oh SJ, Lee EK, Hur NH, Park JT (2005). Phase- and size-controlled synthesis of hexagonal and cubic CoO nanocrystals. J Am Chem Soc.

[34] Lim WP, Zhang ZH, Low HY, Chin WS (2004). Preparation of Ag_2_S nanocrystals of predictable shape and size. Angew Chem Int Ed.

[35] Wang XB, Liu WM, Hao JC, Fu XG, Xu BS (2005). A simple large-scale synthesis of well-defined silver sulfide semiconductor nanoparticles with adjustable size. Chem Lett.

[36] Dong LH, Chu Y, Liu Y (2008). Synthesis of faceted and cubic Ag_2_S nanocrystals in aqueous solution. J Colloid Interf Science.

[37] Sun YZ, Zhou BB (2010). Single-crystalline Ag_2_S hollow nanohexagons and their assembly into ordered arrays. Mater Lett.

[38] Zhuang ZB, Peng Q, Zhang B, Li YD (2008). Controllable synthesis of Cu_2_S nanocrystals and their assembly into superlattice. J Am Chem Soc.

[39] Zhuang Z, Peng Q, Wang X, Li Y (2007). Tetrahedral colloidal crystals of Ag_2_S nanocrystals. Angew Chem Int Ed.

[40] Chaudhuri RG, Paria S (2012). A novel method for the templated synthesis of Ag_2_S hollow nanospheres in aqueous surfactant media. J Colloid Interf Science.

[41] Liu MY, Xu ZL, Li BN, Lin CM (2011). Synthesis of worm-like Ag_2_S nanocrystals in W/O reverse microemulsion. Mater Lett.

[42] Brelle MC, Zhang JZ, Nguyen L, Mehra RK (1999). Synthesis and ultrafast study of cysteine- and glutathione-capped Ag_2_S semiconductor colloidal nanoparticles. J Phys Chem A.

[43] Ortega EV, Berk D (2006). Precipitation of silver powders in the presence of ethylenediamine tetraacetic acid. Ind Eng Chem Res.

[44] Lv LY, Wang H (2014). Ag_2_S nanorice: hydrothermal synthesis and characterization study. Mater Lett.

[45] Yu C, Ming ML, Liu Z, Yu Y (2012). Synthesis of normal and flattened rhombic dodecahedral Ag2S particles. Cryst Eng Comm.

[46] McGrath KM (2001). Probing material formation in the presence of organic and biological molecules. Adv Mater.

[47] Cai W, Shin DW, Chen K, Gheysens O, Cao Q, Wang SX (2006). Peptide-labeled near-infrared quantum dots for imaging tumor vasculature in living subjects. Nano Lett.

[48] Kong Y, Chen J, Gao F, Li W, Xu X, Pandoli O (2010). A multifunctional ribonuclease-A-conjugated CdTe quantum dot cluster nanosystem for synchronous cancer imaging and therapy. Small.

[49] Yarema M, Pichler S, Sytnyk M, Seyrkammer R, Lechner RT, Fritz-Popovski G (2011). Infrared emitting and photoconducting colloidal silver chalcogenide nanocrystal quantum dots from a silylamide-promoted synthesis. ACS Nano.

[50] Du Y, Xu B, Fu T, Cai M, Li F, Zhang Y (2010). Near-infrared photoluminescent Ag2S quantum dots from a single source precursor. J Am Chem Soc.

[51] Chen J, Zhang T, Feng LL (2013). Synthesis of ribonuclease-A conjugate Ag_2_S quantum dots clusters via biomimetic route. Mater Lett.

[52] Siva C, Chandrasekaran Nivedhini I (2014). L-cysteine assisted formation of mesh like Ag_2_S and Ag_3_AuS_2_ nanocrystals through hydrogen bonds. Mater.Lett.

[53] Koneswaran M, Narayanaswamy R (2009). L-cysteine-capped ZnS quantum dots based fluorescence sensor for Cu^2+^ ion. Sens Actuator B Chem.

[54] de la Rica R, Velders AH (2011). Biomimetic crystallization of Ag_2_S nanoclusters in nanopore assemblies. JAm Chem Soc.

[55] Brennan JG, Siegrist T, Carroll PJ, Stuczynski SM, Brus LE, Steigerwald ML (1989). The preparation of large semiconductor clusters via the pyrolysis of a molecular precursor. J. Am. Chem. Soc..

[56] Fan D, Afzaal M, O'Brien P (2007). Using coordination chemistry to develop new route to semiconductor and other materials. Coord Chem Rev.

[57] Esteves ACC, Trindade T (2002). Synthesis studies on II/VI semiconductor quantum dots. Curr Opin Solid State Mater Sci.

[58] Tang Q, Yoon SK, Yang HJ (2006). Selective degradation of chemical bonds: from single source molecular precursors to metallic Ag and semiconducting Ag_2_S nanocrystals via instant thermal activation. Langmuir.

[59] Wang TX, Xiao H, Zhang YC (2008). Simple solid state synthesis of Ag_2_S crystallites using a single source molecular precursor. Mater Lett.

[60] Zhang CL, Zhang SM, Yu LG, Zhang ZJ (2012). Size-Controlled synthesis of monodisperse Ag_2_S nanoparticles by a solventless thermolytic method. Mater Lett.

[61] Hou XM, Zhang XL, Yang W, Liu Y (2012). Synthesis of SERS active Ag_2_S nanocrystals using oleylamine as solvent reducing agent and stabilizer. Mater Res Bull.

[62] Shakouri-Arani M, Salavati-Niasari M (2014). Structural and spectroscopic characterization of prepared Ag_2_S nanoparticles with a novel sulfuring agent. Mol Biomol Spectrosc.

[63] Wehrenberg BL, Wang C, Guyot-Sionnest P (2002). Interband and intraband optical studies of PbSe colloidal quantum dots. J Phys Chem B.

[64] Bakueva L, Gorelikov I, Musikhin S, Zhao XS, Sargent EH, Kumacheva E (2004). PbS quantum dots with stable efficient luminescence in the near-IR spectral range. Adv Mater.

[65] Harrison MT, Kershaw SV, Burt MG, Eychmuller A, Weller H, Rogac AL (2000). Wet chemical synthesis and spectroscopic study of CdHgTe nanocrystals with strong near-infrared luminescence. Mater Sci Eng B.

[66] Zrazhevskiy P, Senawb M, Gao X (2010). Designing multifunctional quantum dots for bioimaging, detection, and drug delivery. Chem Soc Rev.

[67] O'Connell MJ, Bachilo SM, Huffman CB, Moore VC, Strano MS, Haroz EH, Rialon KL, Boul PJ, Noon WH, Kittrell C (2002). Band gap fluorescence from individual single-walled carbon nanotubes. Science.

[68] Crochet J, Clemens M, Hertel T (2007). Quantum yield heterogeneities of aqueous single-wall carbon nanotube suspensions. J Am Chem Soc.

[69] Zhang Y, Hong GS, Zhang YJ (2012). Ag_2_S quantum dot: a bright and biocompatible fluorescent nanoprobe in the second near-infrared window. ACS Nano.

[70] Jiang P, Zhu CN, Zhang ZL (2012). Water-soluble Ag_2_S quantum dots for near-infrared fluorescence imaging in vivo. Biomaterials.

[71] Zhou XD, Shi HQ, Huang DM, Jia SM (2008). Room temperature synthesis and electrochemical application of imidazoline surfactant-modified Ag_2_S nanocrystals. Mater Lett.

[72] Sohrabnezhad S, Pourahmad A (2010). Comparison absorption of new methylene blue dye in zeolite and nanocrystal zeolite. Desalination.

[73] Pourahmad A, Pourahmad A, Sohrabnezhad S, Rakhshaee R (2011). Ternary metal sulphide nanocrystals in MCM-41 nanoparticles matrix: preparation and properties. Micro Nano Lett.

[74] Aazam ES (2014). Photocatalytic oxidation of methylene blue dye under visible light by Ni doped Ag_2_S nanoparticles. J. Ind. Eng. Chem.

[75] Pourahmad A (2012). Ag_2_S nanoparticle encapsulated in mesoporous material nanoparticles and its application for photocatalytic degradation of dye in aqueous solution. Superlattices and Microst.

[76] Robert D (2007). Photosensitization of TiO_2_ by M_x_O_y_ and M_x_S_y_ nanoparticles for heterogeneous photocatalysis applications. Catal Today.

[77] Kim JC, Choi J, Lee YB, Hong JH, Lee JI, Yang JW, Lee WI, Hur NH. Enhanced photocatalytic activity in composites of TiO_2_ nanotubes and CdS nanoparticles. Chem. Commun*.* 2006, 5024–5026. Epub 2006 Oct 27. 10.1039/b612572g 17146515

[78] Puddu V, Mokaya R, Puma GL. Novel one step hydrothermal synthesis of TiO_2_/WO_3_ nanocomposites with enhanced photocatalytic activity.Chem. Commun. 2007, 4749–4751. Epub 2007 Sep 7. 10.1039/b711559h 18004429

[79] Neves MC, Nogueira JMF, Trindade T, Mendonca MH (2009). Photosensitization of TiO_2_ by Ag_2_S and its catalytic activity on phenol photodegradation. J Photochem Photobiol A Chem.

[80] Shen SH, Guo LJ, Chen XB, Ren F (2010). Effect of Ag_2_S on solar-driven photocatalytic hydrogen evolution of nanostructured CdS. Int J Hydrogen Energy.

[81] Mo ZL, Liu PE, Gou RB, Deng ZP (2012). Graphene Sheets/Ag_2_S nanocomposites: synthesis and their application in supercapacitor materials. Mater Lett.

[82] Marti A, Araujo GL (1996). Limiting efficiencies for photovoltaic energy conversion in multigap systems. Sol Enrgy Mater Sol Cells.

[83] Auttasit T, Wu KL, Tung HY (2010). Ag_2_S quantum dot-sensitized solar cells. Electrochem Commun.

[84] Karbmzadeh R, Aleali H, Mansour N (2011). Thermal nonlinear refraction properties of Ag2S semiconductor nanocrystals with its application as a low power optical limiter. Opt Commun.

[85] Xu MY, Niu HL, Huang JJ, Song JM, Mao CG, Zhang SY, Zhu CF, Chen CL (2015). Facile synthesis of graphene-like Co_3_S_4_ nanosheet/Ag_2_S nanocomposite with enhanced performance in visible light photocatalysis. Appl Surf Sci.

[86] Li ZH, Shen J, Wang JQ, Wang DJ, Huang YJ, Zou J (2012). Single crystal titanate–zirconate nanoleaf: synthesis, growth mechanism and enhanced photocatalytic hydrogen evolution properties. Cryst Eng Comm.

[87] Sun YF, Sun ZH, Gao S, Cheng H, Liu QH, Lei FC, Wei SQ, Xie Y (2014). All-surface-atomic-metal chalcogenide sheets for high-efficiency visible light photoelectrochemical water splitting. Adv Energy Mater.

[88] Gubicza A, Csontos M, Halbritter A, Mihaly G (2015). Non-exponential resistive switching in Ag_2_S memristors: a key to nanometer-scale non-volatile memory devices. Nanoscale.

[89] Nasrallah TB, Dlala H, Amlouk M, Belgacem B (2005). Some physical investigations on Ag_2_S thin films prepared by sequential thermal evaporation. Synth Met.

[90] Karashanova D, Nihtianova D, Starbov K (2004). Crystalline structure and phase composition of epitaxially grown Ag_2_S thin films. Solid State lonics.

[91] Hamilton JF (1988). The silver halide photographic process. Adv Phys.

[92] Karashanova D, Starbov N (2006). Surface assisted electric transport in Ag_2_S thin films. Appl Surf Sci.

[93] Kuhl M, Steuckart C, Eickert G, Jeroschewski P (1998). A H_2_S microsensor for profiling biofilms and sediments: application in an acidic lake sediment. Aquat Microb Ecol.

[94] Ding K, Seyfried WE, Tivey MK, Bradley AM (2001). In situ measurement of dissolved H_2_ and H_2_S in high temperature hydrothermal vent fluids at the Main Endeavour Field, Juan de Fuca Ridge. Earth Planet Sci Lelt.

[95] Zhang RH, Zhang XT, Hu SM (2013). Novel sensor based on Ag/Ag_2_S electrode for in situ measurement of dissolved H_2_S in high temperature and pressure fluids. Sens Actuators B Chem.

[96] Ding Q, Pan YW, Huang YF. The optimization of Ag/Ag_2_S electrode using carrier electroplating of nano silver particles and its preliminary application to offshore Kueishan Tao, Taiwan. Continental Shelf Research. 2015, 25

